# Lateral Pressure Application to the Seated Buttocks Largely Maintains Transcutaneous Gas Tensions at the Ischial Tuberosity in a Healthy Cohort

**DOI:** 10.1111/iwj.71054

**Published:** 2026-09-24

**Authors:** Maegan Spiteri, Louise Savine, Peter R. Worsley, Spyros Masouros

**Affiliations:** ^1^ Department of Bioengineering Imperial College London London UK; ^2^ Tissue Viability Imperial College Healthcare NHS Trust London UK; ^3^ Skin Sensing Research Group, School of Health Sciences University of Southampton Southampton UK

**Keywords:** perfusion, pressure ulcer, primary prevention, sitting position, transcutaneous oximetry

## Abstract

This study aimed to evaluate the physiological effectiveness of lateral pressure application in maintaining baseline transcutaneous oxygen (TcPO_2_) and carbon dioxide (TcPCO_2_) tensions in the buttocks during sitting, compared with a conventional static foam cushion. Secondary aims were to determine whether body mass index (BMI) influenced the physiological response when lateral pressure was applied and to investigate the relationship between transcutaneous oximetry and underbody interface pressure. A prospective, controlled, repeated‐measures laboratory study was conducted with 30 healthy adults. Participants underwent two 20‐min sitting conditions: sitting on static foam alone and sitting on static foam with lateral pressure applied by a prototype device. Transcutaneous gas tensions were measured at the right ischial tuberosity using an oximeter and normalised to unloaded standing baselines. Tissue responses were categorised according to the Chai and Bader tissue‐response criteria, where changes in gas tensions of < 25% are Category 1, least risk, and changes of > 25% are Category 3, highest risk. Underbody interface pressures were recorded using a pressure‐mapping array. Statistical comparisons between conditions were performed using paired parametric or non‐parametric tests as appropriate. Mean and peak underbody interface pressures were significantly reduced (*p* < 0.0001) when lateral pressure was applied. Lateral pressure significantly reduced the mean change in TcPCO_2_ compared to static foam alone (11.9% ± 25.6% and 47.8% ± 72.9%, respectively, *p* < 0.0001) and reduced the change in TcPO_2_ to −29.5% ± 23.5% compared to −50.4% ± 34.8% with static foam alone (*p* < 0.0001). Eleven participants exhibited a Category 3 ischaemic response on static foam, compared with only two when lateral pressure was applied. Twenty‐nine of 30 participants moved to a more favourable or unchanged tissue‐response category when lateral pressure was applied. No correlation was found between interface pressure and changes in gas tensions. No significant differences were observed between BMI groups. Application of lateral pressure during sitting maintained tissue perfusion at the ischial tuberosity more effectively than static foam alone. These findings support the physiological rationale for applying lateral pressure as a novel strategy for pressure‐ulcer prevention in seated patients.

## Key Points

- Applying lateral pressure during sitting maintains transcutaneous gas tensions at the ischial tuberosity significantly closer to unloaded baseline, compared to a static foam chair alone.
- Applying lateral pressure reduced the number of people experiencing a high‐risk category 3 tissue‐response (as defined by Chai and Bader) to 2/30 people, compared to 11/30 with the static foam chair alone.
- The lateral pressure intervention significantly reduced mean and peak interface pressure, as well as peak pressure gradient for all participants compare to the static foam chair alone.
- There was found to be no statistical difference in tissue response during sitting between those with BMI < 25 kg m^−2^ and BMI > 25 kg m^−2^.

## 1 Introduction

Pressure ulcers (PUs), also known as pressure injuries or bed sores, are painful and incapacitating wounds that commonly develop in individuals with prolonged immobility, particularly those confined to beds or wheelchairs [1]. These injuries typically occur over bony prominences that are in contact with support surfaces, frequently at anatomical sites like the sacrum and ischial tuberosities in the buttocks [2, 3]. The burden of PUs on patient well‐being is substantial, prompting major healthcare spending on prevention and treatment alike [4]. Current evidence suggests that the global prevalence of PUs among hospitalised patients is approximately 12%, with some studies from the United States recording incidence rates as high as 40% [5]. In England, it is estimated that there are approximately 2000 new PU cases each month, costing its National Health Service (NHS) approximately £3.8 million each day [6]. A large study of PU risk factors in people with mobility‐related disabilities found that 7% of participants had at least one PU [7]. Of those with PUs, 82% reported ulcers in the pelvic or buttock region, highlighting the burden of seating‐acquired PUs.

International best‐practice guidelines for PU prevention recommend that adults at risk are regularly repositioned when sitting or lying down, with clinicians advised to use professional judgement when identifying turning frequency based on individual factors such as tissue integrity, patient mobility and overall comfort [8]. However, multiple systematic reviews have reported insufficient evidence to determine conclusively the most effective repositioning intervals or specific body positions, reflecting ongoing uncertainty and debate in clinical practice [9, 10, 11, 12, 13, 14].

Clinical guidelines also support the use of pressure‐redistributing support surfaces, including specialised mattresses, seat cushions and therapeutic chairs, for individuals in hospitals, or for those at increased risk of developing PUs in community settings [1, 15]. For seated individuals, static‐foam cushions are commonly used to distribute pressure more evenly across the buttocks, while patients considered to be at higher risk are often prescribed more advanced technologies, such as air‐based systems with alternating pressure (AP) functionality [16]. Although these technologies are prescribed routinely with the intent of reducing risk in seated patients, the overall body of supporting evidence remains weak [16, 17, 18, 19, 20, 21]. Furthermore, there is growing recognition that interface pressure, a commonly used metric to evaluate support surfaces, does not reliably predict PU incidence [22, 23, 24, 25]. The substantial financial investment in these devices, without conclusive data, highlights the need for both innovation in device design and high‐quality evidence to demonstrate their clinical value.

**FIGURE 1:**
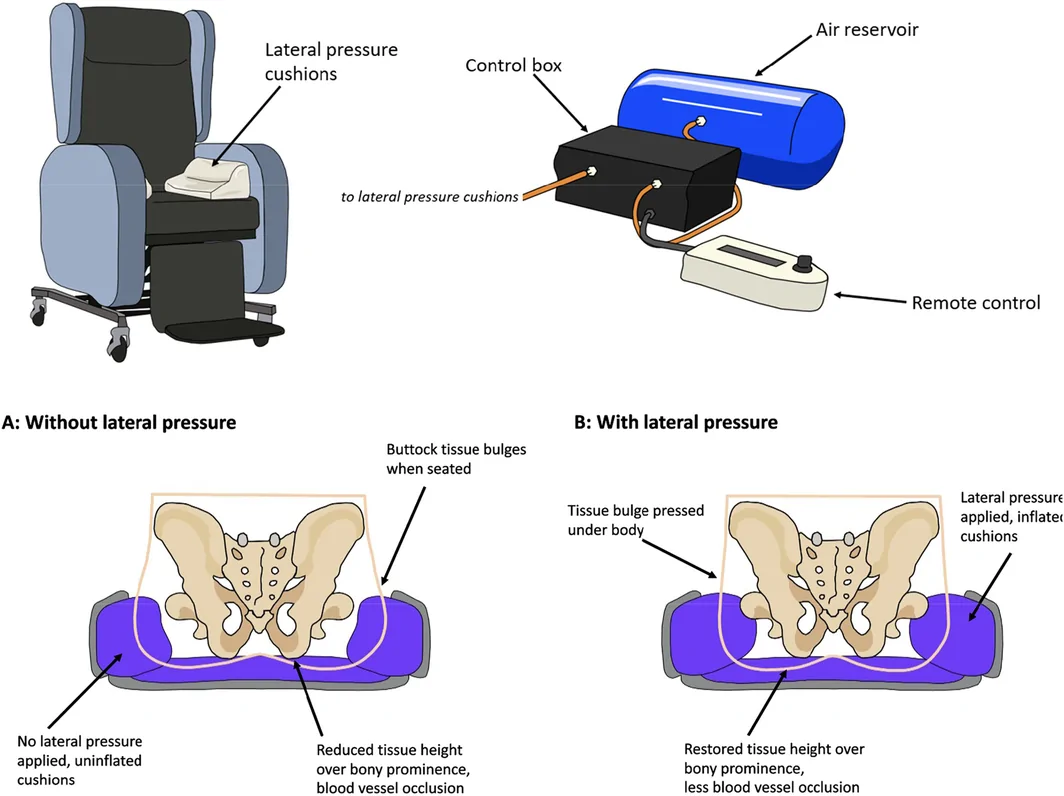
Top: Diagram of the lateral pressure (LP) device. Bottom: Diagram summarising the effect of applying lateral pressure to the seated buttock.

The authors recently introduced a novel approach involving lateral pressure (LP) application as a method to mitigate PU risk [26, 27, 28]. The laboratory prototype system to apply LP uses air‐filled bladders positioned against the sides of an armchair which can inflate to apply pressure inwards, on the user's thighs and buttocks (Figure 1 *top*). This pressure gently pushes the bulged soft tissue of the buttocks back underneath the seated individual, effectively increasing the tissue height over vulnerable bony prominences such as the ischial tuberosities, as well as helping to preserve blood flow by reducing vascular compression [26] (Figure 1 *bottom*). Preliminary work determined the optimum amount of lateral pressure necessary to tissue perfusion at the IT without compromising tissue health at the greater trochanter (GT) and showed the potential benefits of LP application in a small heterogenous sample of eight healthy volunteers [26]. The present study follows from this work to show the effect of lateral pressure application across a larger cohort of 30 participants.

The version of the device used in previous studies included an underbody cushion, with its internal pressure being a surrogate measure of the seated individual's weight [26, 28]. For simplicity of design, this has been removed, and instead the amount of lateral pressure to be applied is determined by inputting the participant's weight into the system, and is equivalent to application of a 50% ratio of lateral‐to‐underbody pressure, described in previous work [26, 28].

This study aims to evaluate the effectiveness of the new version of the lateral pressure device in maintaining baseline transcutaneous oxygen (TcPO_2_) and carbon dioxide (TcPCO_2_) tensions within the buttocks tissue, compared to a conventional static foam cushion, in a cohort of healthy individuals. Transcutaneous oximetry has been used widely in PU research to measure gas concentrations in the buttock tissue as a surrogate measure of tissue ischaemia [26, 28, 29, 30, 31, 32, 33]. For example, Worsley et al. used oximetry to evaluate different postures during sitting [34]. They found leaning to the right to resulted in the largest change in transcutaneous gas tensions. Their results also revealed correlations between seated interface peak pressure index and the percentage change in TcPO_2_ (negative trend) and TcPCO_2_ (positive trend).

Secondary aims of this study were to identify whether there is a range of BMI for which application of lateral pressure is consistently beneficial, and to investigate the relationship between transcutaneous oximetry and underbody interface pressure. To achieve these aims, the objectives of this study are:

- To measure and compare transcutaneous gas during periods of sitting with and without the application of lateral pressure.
- To stratify and analyse these gas tensions based on participant BMI.
- To correlate transcutaneous oximetry measurements with underbody interface pressure data.

## 2 Methods

This is a prospective, controlled, repeated‐measures laboratory study in healthy adults. Ethical approval for the study was granted by the [Institutional] Research Ethics Committee (ID 6895421). Thirty healthy participants were recruited who gave informed consent to participate. Participants were required to be 18 years of age or older. Exclusion criteria included any history of PUs or any period of being restricted to a bed or wheelchair in the past 12 months. For this study 30 participants were recruited and attended the laboratory maintained at 22°C. Sample size estimation was informed by the authors' previous pilot study of eight participants using a paired, within‐subject design [28]. For TcPCO_2_, the pilot data demonstrated a mean paired difference of 81 units (SD 103), corresponding to a minimum required sample size of 13 participants for 80% power at a two‐tailed *α* of 0.05. For TcPO_2_, the pilot data demonstrated a mean paired difference of 40 units (SD 34), corresponding to a required sample size of approximately 6 participants under the same assumptions. To account for the small pilot sample size and potential overestimation of effect sizes, the target sample size was conservatively increased to 30 participants.

Before attending, participants were asked to remove hair from the test area on their right buttock as body hair can prevent the oximeter from obtaining accurate readings. Participants wore loose fitting shorts to allow the sensor to be placed easily. Once seated, the footrest of the armchair was adjusted to ensure that the participants' thighs were parallel to the seat base to prevent increased loads through the ischial tuberosities [35]. The participant's age, sex, height, weight, skeletal muscle and body fat were recorded using calibrated tape measure and smart scales (ES‐CS20M, RENPHO, resolution 0.05 kg). The participants had a mean age of 28.9 years (±standard deviation, 9.4 years) and mean BMI 25.5 ± 4.8 kg m^−2^ (Table 1).

**TABLE 1:** Summary of participants and characteristics as a complete sample group (*n* = 30) and stratified by BMI group (BMI < 25 kg m^−2^ *n* = 16, BMI > 25 kg m^−2^ *n* = 14).

Variables	Mean and standard deviation					
	Sample *n* = 30		BMI < 25 kg m^−2^		BMI > 25 kg m^−2^	
Sex (M, F)	16	14	6	10	10	4
Age (years)	28.9	±9.4	24.8	±2.4	33.5	±12.1
Weight (kg)	76.3	±16.5	65.5	±10.3	88.7	±13.1
Height (cm)	172.6	±9.7	170.5	±10.5	175.0	±8.4
BMI (kg m^−2^)	25.5	±4.8	22.3	±1.8	29.1	±4.5
Body fat (%)	27.6	±6.0	24.7	±4.1	30.9	±6.2
Skeletal muscle (%)	44.9	±5.3	45.8	±4.5	43.8	±6.1
Muscle:fat ratio	1.7	±0.5	1.9	±0.6	1.5	±0.5

**FIGURE 2:**
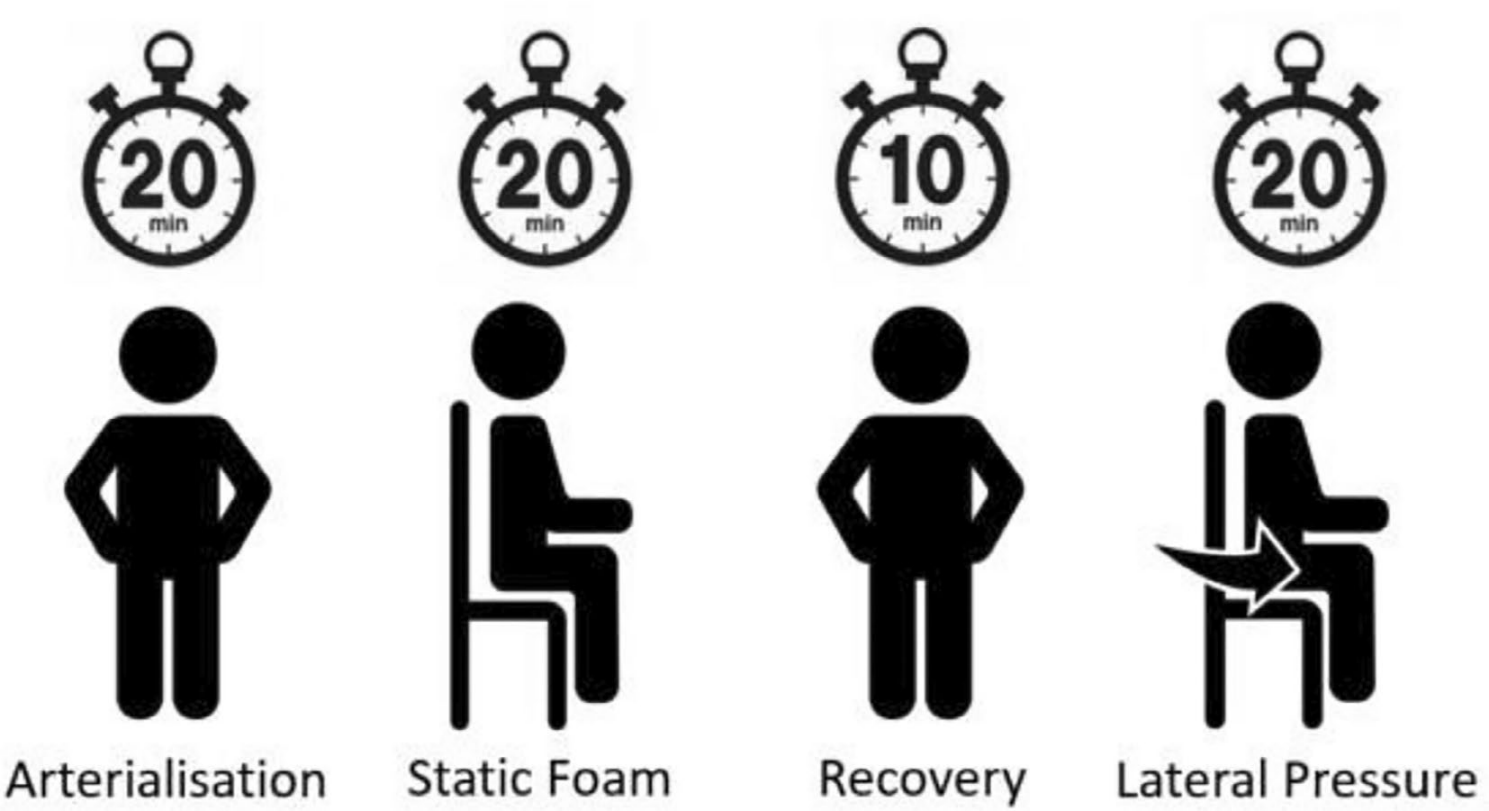
Pictorial summary of testing sequence.

Prior to the attendance of each participant, the lateral pressure device was set up on a static‐foam therapeutic armchair, and the oximeter (resolution 0.1 mmHg, sampling frequency 1 Hz, O_2_ range 0–800 mmHg, CO_2_ range 5–200 mmHg; Radiometer TCM5 Flex, Denmark) was calibrated as per its user manual. A soft 16 × 16 pressure‐mapping array (resolution 0.1 kPa, sampling frequency 5 Hz, range 2.6–26.6 kPa; Model SVZE4545L, SR SoftVision, Japan) was placed over the seat of the chair and connected to a laptop running the mapping software. With verbal guidance from the investigator to explain how to locate the bone, the participants fit their own oximetry electrodes with a fixation ring at their right ischial tuberosity then underwent the following testing regime (Figure 2).

- Stand for 20 min (during which the sensor heated the skin to ensure maximum vasodilation).
- Sit for 20 min on static foam.
- Stand for 10 min to allow gas tensions to return to unloaded baseline level.
- Sit for 20 min with lateral pressure applied.

During sitting periods, the participants were asked to avoid resting their arms on the arm rests of the chair to ensure all body weight was carried consistently by the pelvis. Finally, participants completed a questionnaire about their exercise levels, and how they experienced application of lateral pressure.

The mean change in gas tensions between 4 and 9 min during each standing period was calculated to establish the standing control baseline, to which data from each corresponding seated condition were normalised (calculation of percentage change between standing and sitting). Additionally, the mean change in gas tensions was calculated over 10–19 min during all sitting periods. These specific timeframes were chosen based on preliminary findings suggesting that most individuals reached a plateau in their gas tensions within 5–10 min. Changes in gas tensions were categorised according to the established characteristic responses defined by Chai and Bader [29] namely:

- Category 1: minimal changes in both TcPO_2_ and TcPCO_2_ values.
- Category 2: > 25% decrease in TcPO_2_ with minimal change in TcPCO_2_.
- Category 3: > 25% decrease in TcPO_2_ associated with a > 25% increase in TcPCO_2_.

Category 1 is the safest and therefore most desirable tissue state, whereas Category 3 is associated with full ischemia of microvascular structures, presenting a risk of tissue damage which should be avoided [29].

Underbody pressure data was exported and processed using MATLAB (version 23.2 (R2023b), MathWorks) to calculate the mean and peak underbody pressure for each participant for 5–15 min of each 20‐min sitting period. To evaluate spatial pressure distribution and localised tissue deformation, peak pressure gradients (PPG) were calculated for each frame using established methods [36]. Prior to gradient calculations, a 3 × 3 moving average spatial filter was applied across the 16 × 16 sensor grid to smooth noise. Sensor arrays were split into left (*x*‐coordinates 2–8) and right (*x*‐coordinates 9–15) anatomical regions across rows 2–15 to assess each ischial tuberosity independently. For each interior sensor, pressure differentials were calculated against its four orthogonal adjacent sensors (distance *d* = 3.0 cm) and four diagonal adjacent sensors (distance *d* = 3.0√2 cm). The maximum pressure derivative per sensor was defined as its local gradient. Frame‐wise PPGs were extracted across 5–15 min for each sitting period and averaged to yield representative left‐ and right‐sided PPG values for each participant and sitting condition.

Statistical analysis was performed using GraphPad Prism (v10.3.1 for Windows, GraphPad Software). Data normality was assessed to determine appropriate parametric or non‐parametric tests. Paired two‐tailed Student's *t*‐tests were used to evaluate bilateral differences in PPG within both the static sitting and lateral pressure conditions. Paired one‐tailed tests were used to compare changes in pressure parameters, PPG and oximetry metrics between the static sitting and lateral pressure conditions. One‐tailed tests were chosen as previous work by the authors already established that application of lateral pressure improves oximetry. Correlations between PPG and changes in transcutaneous gas tensions were evaluated using Pearson's *r* or Spearman's rank correlation coefficients based on data distribution. Participants were split into two groups depending on BMI, using the World Health Organisation's classification; Group 1 were underweight or healthy weight (BMI < 25 kg m^−2^), and Group 2 were overweight or obese (BMI > 25 kg m^−2^) [37]. Statistical analysis comparing the two groups was carried out using an unpaired, two‐tailed Student's *t*‐test.

## 3 Results

**FIGURE 3:**
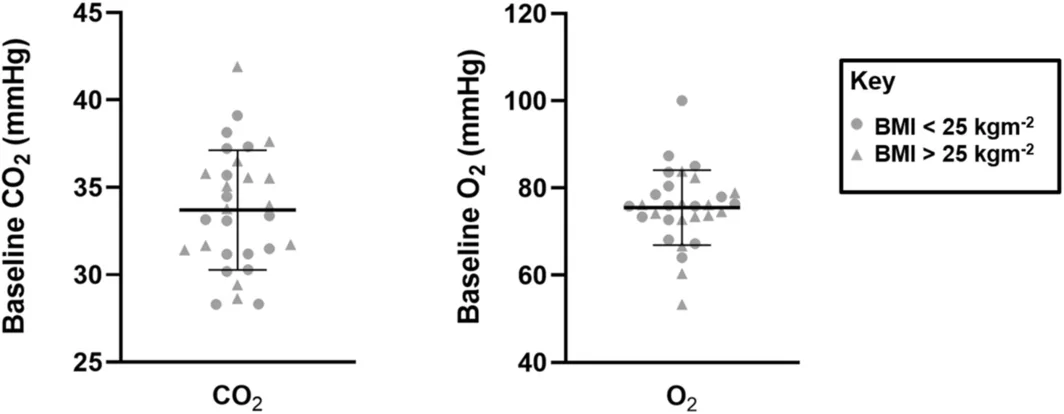
Baseline gas tensions of carbon dioxide (left) and oxygen (right) of all 30 participants in mmHg where circles and triangles are participants with BMI < 25 kg m^−2^, and BMI > 25 kg m^−2^, respectively. There is no statistical difference in baseline gas tensions of carbon dioxide or oxygen between those with BMI < 25 kg m^−2^ and BMI > 25 kg m^−2^.

Figure 3 shows the baseline gas tensions of carbon dioxide and oxygen for the 30 participants. The mean (±1 standard deviation) baseline carbon dioxide and oxygen concentrations were 33.7 ± 3.42 mmHg and 75.5 ± 8.62 mmHg, respectively. For baselines carbon dioxide, the mean concentrations were 33.3 ± 3.43 mmHg for BMI < 25 kg m^−2^ and 34.6 ± 3.33 mmHg for BMI > 25 kg m^−2^. For baselines oxygen, the mean concentrations were 77.6 ± 8.69 mmHg for BMI < 25 kg m^−2^ and 74.6 ± 6.08 mmHg for BMI > 25 kg m^−2^. There was found to be no statistical difference in baseline gas tensions of carbon dioxide or oxygen between those with BMI < 25 kg m^−2^ and BMI > 25 kg m^−2^, with *p* values 0.48 and 0.15 respectively.

**FIGURE 4:**
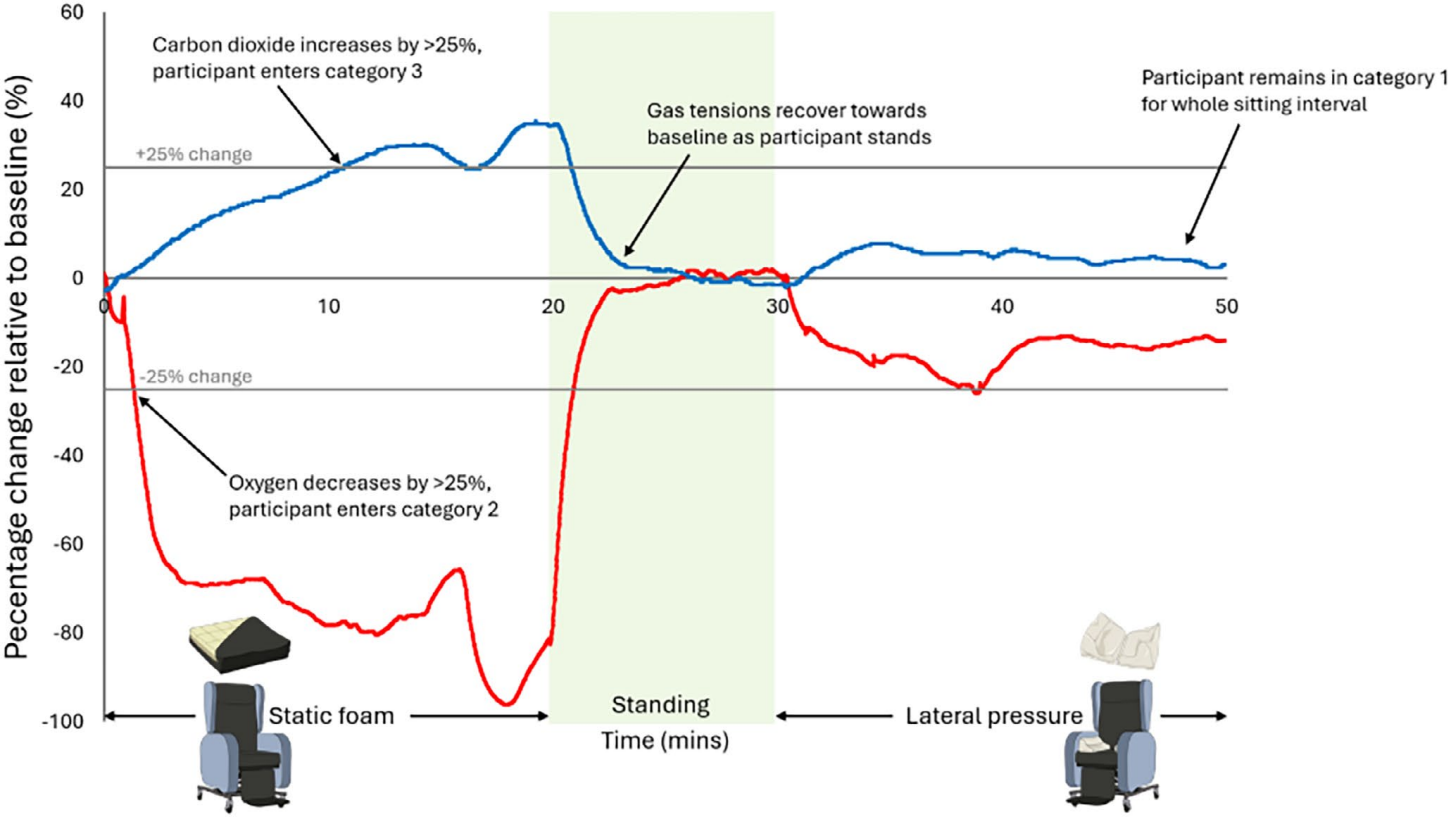
Percentage changes in gas tensions of one female participant while sitting on static foam (20 min), standing (10 min) and sitting on the static foam with lateral pressure (LP) applied (20 min).

The trace of percentage change of gas tensions over time from one participant (female, age 24 years, BMI 20.8 kg m^−2^) is shown in Figure 4. The first sitting period of 20 min on static foam resulted in an increase in CO_2_ and decrease in O_2_ to levels that put this participant in Category 3. The following 10 min of standing brought the gas tensions back to baseline. The second sitting period of 20 min with lateral pressure applied resulted in a milder change compared to the first sitting increase in CO_2_ and decrease in O_2_, which kept the participant within a Category 1 response throughout.

**FIGURE 5:**
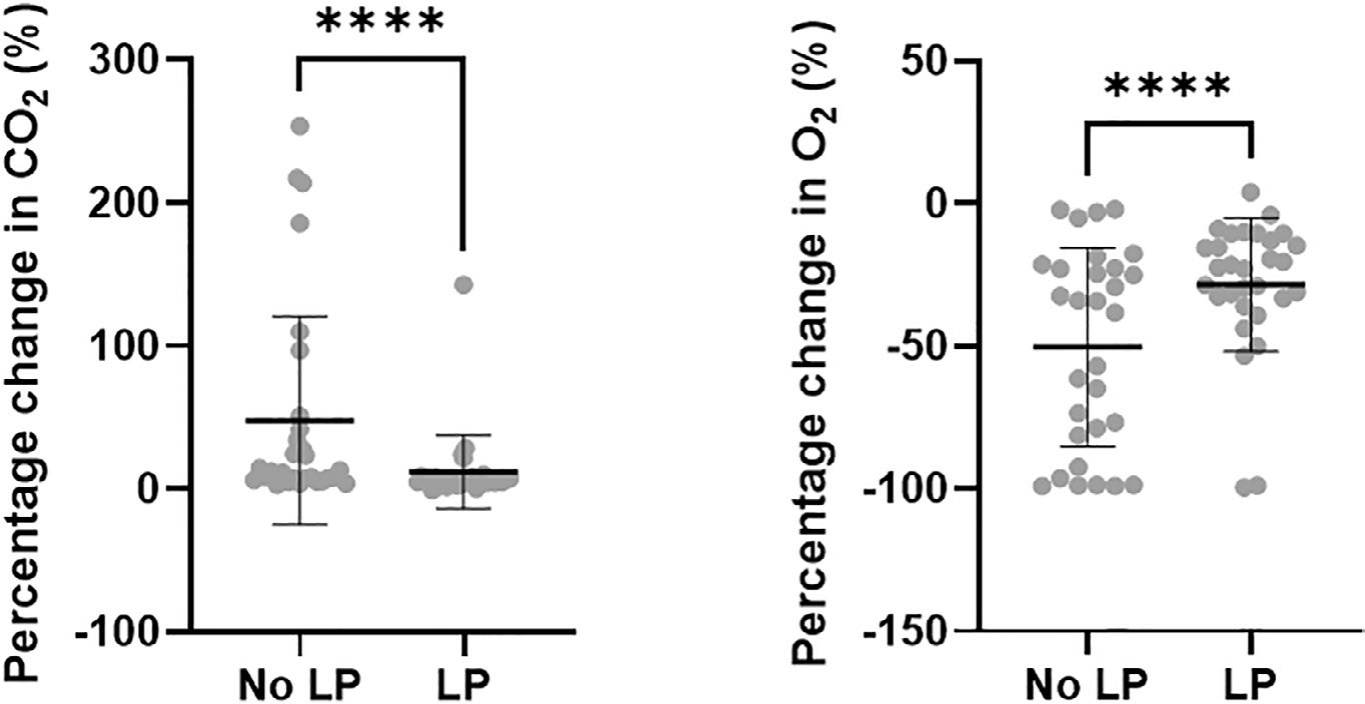
Percentage change of carbon dioxide (left) and oxygen (right) for all participants during sitting periods on static foam and lateral pressure interventions, where **** denotes *p* < 0.0001.

Figure 5 shows the percentage change in gas tensions due to sitting. The mean increase in carbon dioxide was 47.8% ± 72.9% while sitting on the static foam chair. Conversely, the mean increase in carbon dioxide while sitting on the LP device was 11.9% ± 25.6%, demonstrating a statistically significant decrease compared to static foam (*p* < 0.0001). The mean decrease in oxygen was −50.4% ± 34.8% while sitting on the static foam, but only −29.5% ± 23.5% when sitting on the LP device, demonstrating a significantly better ability to maintain baseline oxygen than the static foam (*p* < 0.0001). There was found to be no statistical difference in changes in TcPO_2_ and TcPCO_2_ for both static foam and lateral pressure between those with BMI < 25 kg m^−2^ and BMI > 25 kg m^−2^. Furthermore, there was no statistical difference in BMI between those experiencing a Category 1 and Category 3 response while sitting on static foam alone, nor was there any statistical difference in percentage change in oxygen or carbon dioxide during any period of sitting.

**FIGURE 6:**
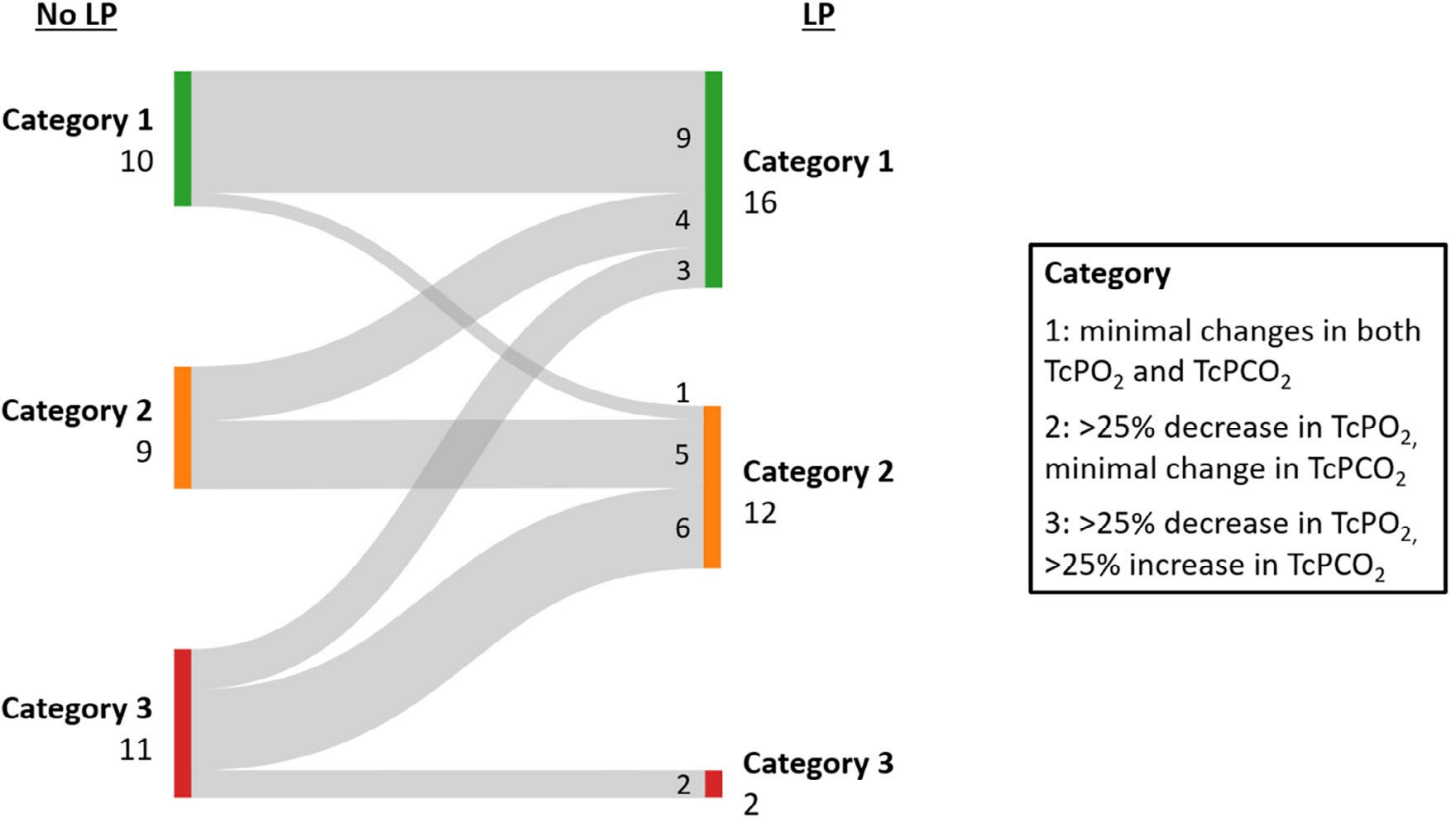
Number of participants in each Chai and Bader category [29] during 20–25 min when sitting on static foam and when lateral pressure was applied.

Figure 6 shows the number of participants in each tissue‐response category during sitting on each support surface. On static foam, 11/30 participants experienced Category 3 ischaemic response, compared to only 2/30 when lateral pressure was applied. When lateral pressure was applied after a period of sitting on static foam, 29/30 participants moved to a safer, more desirable risk category or stayed in the same category. For participants that stayed in the same category after lateral pressure was applied (*n* = 16), all 16 saw a positive change in carbon dioxide levels compared to when sitting on static foam, and 14 saw a positive change in oxygen level. The mean change in gas tensions between sitting on static foam and when lateral pressure applied was a 35.9% ± 66.8% reduction in carbon dioxide level, and a 21.9% ± 25.3% increase in oxygen level.

**FIGURE 7:**
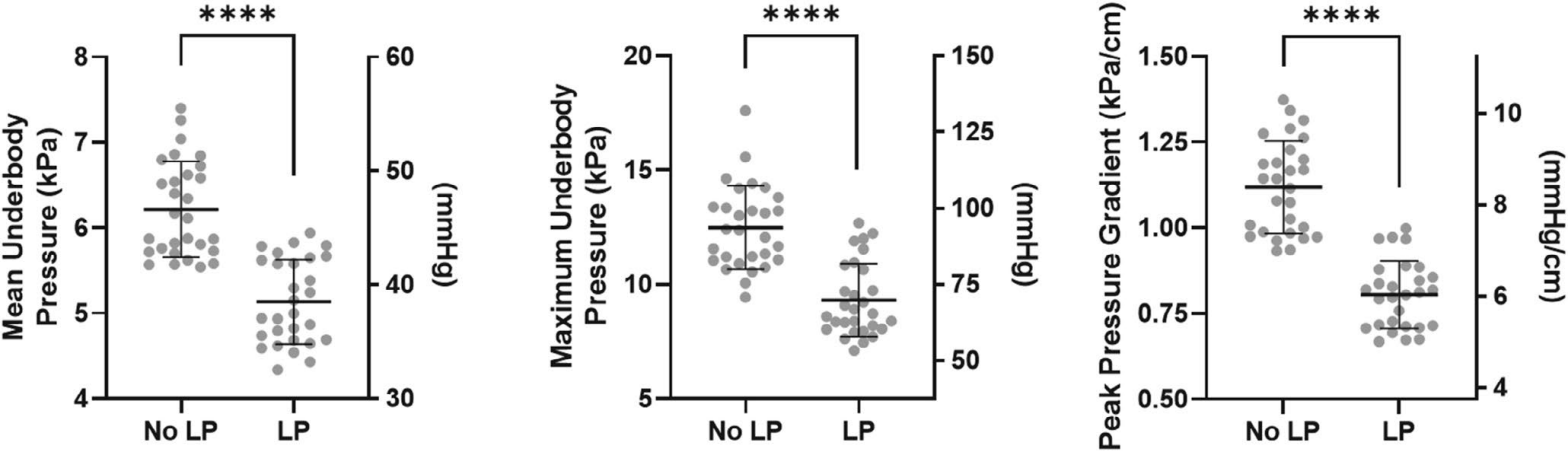
Mean (left), peak (middle) underbody pressures and peak pressure gradient (right) for each participant during sitting on static foam and lateral pressure, where **** denotes *p* < 0.0001.

Underbody pressure data were collected for 93% of participants (*n* = 28) due to data loss (Figure 7). The underbody pressure recorded by the pressure mapping array across participants during sitting on static foam had a mean of 6.2 ± 0.6 kPa, with mean peak pressure 12.6 ± 1.8 kPa. Conversely, when lateral pressure was applied, the mean, and peak underbody pressures were 5.1 ± 0.5 kPa and 9.4 ± 1.6 kPa, respectively, demonstrating a statistically significant decrease compared to sitting on static foam (*p* < 0.0001). PPG was calculated for the left side of the buttocks to remove the effect of the oximetry sensor at the right IT, however, there was found to be no significant difference between PPG on the left side and right side of the buttocks during either seating condition. The mean PPG of participants sitting on static foam was 1.1 ± 0.1 kPa/cm compared to 0.8 ± 0.1 kPa/cm during sitting with lateral pressure. This is a statistically significant reduction in PPG due to the application of lateral pressure (*p* < 0.0001). Every participant experienced a reduction in mean and peak underbody pressure, and PPG, regardless of change in transcutaneous gas tensions. In addition, there was found to be no statistically significant correlation between underbody pressure and change in gas tensions, nor any difference in underbody pressure between those experiencing Category 1 or Category 3 during static foam sitting. There was also no correlation between PPG and change in gas tensions.

Participants were asked questions about their lifestyle, including how many hours per day they spent sitting, and the types and frequency of exercise. There was found to be no correlation between these parameters and baseline gas tensions or tissue response when sitting on any of the two configurations tested here.

## 4 Discussion

This study evaluated the physiological efficacy to healthy volunteers of a simple version of a lateral pressure device which aims to prevent seating acquired PUs. The case‐controlled study revealed that the lateral pressure device helped maintain tissue perfusion during periods of sitting, with a significantly better outcome than a standard foam cushion comparator. The results also revealed that interface pressures and PPG were improved with lateral pressure support, confirming the hypothesis that this technology can redistribute pressure and tissue distortion, thus maintain viability of tissue under the vulnerable bony prominence of the ischial tuberosity.

Methods used to compare PU‐prevention devices remain largely unstandardized. Many studies rely on interface pressure as a surrogate marker of efficacy [38, 39, 40], yet there is substantial evidence indicating that interface pressure alone is a poor predictor of PU risk [22, 23, 27]. One key limitation is that it reflects only surface‐level loading and fails to capture the mechanical stresses occurring deeper within the tissue, stresses that are often implicated in the development of deep tissue injuries. In contrast, oximetry has been widely adopted as a more physiologically relevant tool for evaluating the performance of PU‐prevention mattresses [29, 41] and for assessing the impact of seated posture [34, 42, 43, 44]. Oximetry has been shown to indicate reliably tissue compromise in seated individuals, serving as a non‐invasive marker of ischaemia‐induced damage [34, 42, 43, 44, 45]. Prior studies have demonstrated that elevated TcPCO_2_ may signal metabolic disturbances in tissue due to impaired perfusion, offering an early indicator of pressure‐induced damage during mechanical loading [33, 46]. This is particularly relevant in identifying a severe ischaemic response, known as a Category 3 response. As such, both interface pressure and transcutaneous oximetry were used in this study and highlight the beneficial action of lateral pressure.

Application of lateral pressure when sitting resulted in maintaining the transcutaneous gas tensions closer to the unloaded baseline compared to when sitting on static foam, without lateral pressure applied. This finding aligns with results from previous studies [26, 28] and lends support to the underlying theory of the preventative potential of lateral pressure application [27]. Applying pressure from the sides minimises distortion of the soft tissue and upholds its unloaded shape, by preventing lateral displacement of buttock tissue, which results in reduced tissue deformation underneath thus maintaining tissue height over the ITs. This reduces the risk of blood‐vessel occlusion, thereby mitigating the changes in oxygen and carbon dioxide concentrations associated with tissue ischaemia. Specifically, 11 participants experienced a Category 3 response when sitting without lateral pressure applied. This reduced to just two participants when lateral pressure was applied, who, despite remaining in Category 3, saw a sizeable reduction in the carbon dioxide concentration when lateral pressure was applied.

One individual experienced a significantly different increase in carbon dioxide with LP compared to the rest of the group. There was no recorded biometric or lifestyle variable for which this person was an outlier to account for this difference. Without LP, this individual saw an increase in carbon dioxide concentration of approximately 186%; with LP they experienced an increase of 143% relative to their baseline measurement. Interestingly, they saw no change in their oxygen concentration under either seating condition, experiencing a decrease of approximately 99% each time. Despite the large deviations both time, lateral pressure did have a small positive effect, but not strong enough to reduce the tissue response category.

Previous work with 17 participants observed that the 3 participants with lowest BMI (20.3–25.0 kg m^−2^) exhibited a Category 3 response, while the other 14 participants (25.0 < BMI < 32.5 kg m^−2^) experienced Category 1 and 2 responses [31]. The present study found no statistical difference in BMI between those experiencing a Category 1 or Category 3 response; this may be due to the larger sample size of 30 compared to 17 previously, or the younger average age of participants. Many studies report an increased PU incidence in those with BMI < 19 or > 40 kg m^−2^ [47, 48, 49]. The present study tested only one participant within each of these BMI brackets. The participant with BMI = 18.7 kg m^−2^ experienced a Category 1 response with both seating interventions, whereas the participant with BMI = 40.9 kg m^−2^ experienced a Category 3 response with static foam, but Category 1 with lateral pressure.

All 30 participants experienced a reduction in mean and maximum underbody interface pressure when sitting with the lateral pressure applied compared to static foam alone. In addition, no correlation was found between participants' underbody pressure and change in gas tensions, or any difference in underbody pressure between those experiencing Category 3 or 1 while sitting on static foam. This finding supports the growing body of evidence that interface pressure is not a robust metric of tissue viability or PU risk and thus should not be used for the evaluation of PU‐prevention devices [22, 23]. Two previous studies have shown that, for a given individual, increasing the load applied to the skin results in predictable changes in gas tensions and interface pressure [50, 51]. Nonetheless, to the authors' knowledge, no published work has investigated whether a similar correlation exists between these measures across a population.

Guidelines often suggest repositioning patients every few hours [15, 52]. Preliminary testing in which a participant remained seated for 4 h, showed no meaningful change in the tissue‐response category beyond the initial 10 min. This plateau effect aligns with established experimental protocols in the field [26, 28, 31, 50, 53]. Therefore, the decision was made to limit each seating condition to 20 min, minimising participant time, while still capturing a realistic tissue response.

Participants self‐positioned the oximetry sensor over their right ischial tuberosity, introducing potential variability due to misplacement. Repeated measurements were not conducted per participant, thus inaccurate sensor placement may have led to measurements not taken at the worst location, exactly at the ITs, but in the vicinity. The comparison conducted here between changes in gas tensions would likely not have been influenced by the location of the sensor. Future investigations should quantify the variability associated with sensor positioning to improve data reliability. Additionally, the oximetry probe was placed specifically at the right ischial tuberosity, so the findings pertain to localised tissue responses at and around that site. As such, benefits observed locally may not translate to the entire seated surface. Future work should incorporate multiple probes to monitor simultaneously both loaded and unloaded tissue regions, allowing for a more comprehensive understanding of each device's systemic effects. This would help determine whether the oximetry results for the static foam and LP device are indicative of broader tissue responses. The use of the oximetry sensor can introduce further pressure points at the fixation point, especially when this point, the ITs, are already a location of peak pressures. This may have led to reported peak pressures being artificially inflated beyond the pressure that would have occured without the presence of the sensor. Future oximetry work should add further mitigations to soften the pressure gradient, reducing the effect of the sensor. However, comparison of PPG between the left and right side of the buttocks show no statistical difference, suggesting the sensor is not altering pressure readings substantially.

The study cohort consisted of healthy, relatively young individuals with limited BMI variation. This homogeneity restricts somewhat the generalisability of the findings, as participants' body composition and buttock morphology may differ significantly from those of individuals at high risk of PUs. The transcutaneous oximetry measurements are physiological markers of tissue perfusion and tissue viability, but not direct indicators of pressure injury. Therefore, while LP was shown to improve tissue perfusion, an explicit correlation with reduction of PU risk cannot be drawn. Future studies should include participants whose anthropometric characteristics more accurately reflect the patient populations most vulnerable to PUs during prolonged seated periods. A large‐scale clinical trial in the future may be able to draw explicit conclusions of the effect of LP on PU incidence tested on individuals at risk of skin damage (elderly, neurological impairment, vascular compromised).

Finally, the lateral pressure device tested in this study was an early‐stage prototype. Its lateral chambers are not commercially available at the time of writing and have not been optimised for consistent pressure delivery, repeatable location of force application and adaptability to different body shapes. Despite these limitations, this study provides compelling evidence that application of lateral pressure is more successful at maintaining transcutaneous gas tensions closer to baseline levels than seating on a static foam cushion. These findings are particularly relevant given the limited innovation in the field of seated support surfaces, where product development has largely focused on material composition rather than active load redistribution. In contrast to the extensive standards and guidance available for support surfaces used for the lying patient, such as ISO 20342, there is dearth of internationally recognised guidelines or testing standards for seating systems intended to protect tissue integrity [54]. The lack of such frameworks may hinder both innovation and objective evaluation of novel technologies. The present findings therefore highlight the need for further development of active seating interventions, alongside the establishment of evidence‐based standards to guide their design, testing and clinical implementation.

## 5 Conclusion

The findings of this study showed that controlled application of lateral pressure to a healthy seated individual is consistently effective at not only reducing underbody pressure by redistributing load over the buttocks, but also and at preventing large changes in transcutaneous gas tensions of oxygen and carbon dioxide. Application of lateral pressure presents a novel way to redistribute pressure over the buttocks; further optimisation of the lab prototype used here to deliver lateral pressure will render the device suitable for pressure‐ulcer prevention in seated patients.

## Funding

The authors have nothing to report.

## Ethics Statement

This study was approved by Imperial College Research Ethics Committee (ICREC, ID 6895421).

## Consent

All participants received a participant information sheet and gave informed consent to take part in the study.

## Conflicts of Interest

The authors declare no conflicts of interest.

## Data Availability

The data that support the findings of this study are available on request from the corresponding author. The data are not publicly available due to privacy or ethical restrictions.
